# Care Strategies for Reducing Hospital Readmissions Using Stochastic Programming

**DOI:** 10.3390/healthcare9080940

**Published:** 2021-07-26

**Authors:** Behshad Lahijanian, Michelle Alvarado

**Affiliations:** Department of Industrial and Systems Engineering, University of Florida, Gainesville, FL 32611, USA; alvarado.m@ufl.edu

**Keywords:** OR in health services, hospital readmission, scenario-based stochastic programming, probabilistic constraints, care strategy

## Abstract

A hospital readmission occurs when a patient has an unplanned admission to a hospital within a specific time period of discharge from an earlier or initial hospital stay. Preventable readmissions have turned into a critical challenge for the healthcare system globally, and hospitals seek care strategies that reduce the readmission burden. Some countries have developed hospital readmission reduction policies, and in some cases, these policies impose financial penalties for hospitals with high readmission rates. Decision models are needed to help hospitals identify care strategies that avoid financial penalties, yet maintain balance among quality of care, the cost of care, and the hospital’s readmission reduction goals. We develop a multi-condition care strategy model to help hospitals prioritize treatment plans and allocate resources. The stochastic programming model has probabilistic constraints to control the expected readmission probability for a set of patients. The model determines which care strategies will be the most cost-effective and the extent to which resources should be allocated to those initiatives to reach the desired readmission reduction targets and maintain high quality of care. A sensitivity analysis was conducted to explore the value of the model for low- and high-performing hospitals and multiple health conditions. Model outputs are valuable to hospitals as they examine the expected cost of hitting its target and the expected improvement to its readmission rates.

## 1. Introduction

Hospital readmissions have drawn increased global attention because they impose tremendous burdens on patients and healthcare systems due to the substantial cost of excess readmissions [1]. A hospital *readmission* occurs when a patient has an unplanned admission to a hospital within a specific time period of discharge from an earlier or initial hospital stay. In recent years, medical professionals and policymakers have started to identify the substantial cost of excess readmissions and the potential to reduce healthcare costs and improve the care quality by eliminating preventable readmissions [2]. The time period for hospital readmission is similar in the United States (U.S.) and European countries such as Denmark and England, generally 30 days [3]. However, there may be slight differences in the period’s definition for example, Germany considers this period from initial admission based on particular situations to identify a second admission [3]. A study of the England National Health Service data over the past decade implies large variations in unplanned readmission rate trends across clinical areas, with some regions experiencing substantial increases [4].

In the U.S., nearly a fifth of patients are rehospitalized within 30 days of discharge [5,6]. Consequently, they are estimated to cost the U.S. Centers for Medicare and Medicaid Services (CMS) $17 to $26 billion annually [7,8]. Although it is not clear what proportion of these readmissions is preventable (estimates range from 9% to 59%), a 10% reduction in hospital readmissions would preserve over $1 billion annually in the U.S. [9]. Rising costs of preventable readmissions led the U.S. Centers for Medicare and Medicaid Services (CMS) to establish the Hospital Readmission Reduction Program (HRRP) [10,11]. HRRP penalizes CMS payments to hospitals with high 30-day readmission rates for six conditions: Acute Myocardial Infarction (AMI), Heart Failure (HF), Pneumonia (PN), Chronic Obstructive Pulmonary Disease (COPD), Total Hip/Knee Arthroplasty (HK), and Coronary Artery Bypass Graft (CABG) surgeries [10]. Based on Kaiser Health News (KHN), approximately 80% of hospitals CMS evaluated for the 2018 Fiscal Year (FY) faced penalties [12]. Some hospital readmissions are avoidable and are associated with low-quality care levels during the patients’ hospital stay or poor arrangement of the discharge process [6,13,14]. However, providing the resources and processes to reduce the probability of readmission typically requires additional financial or resource costs to the hospital. This paper helps hospitals develop care strategies that balance financial costs with effective treatment plans when operating under HRRP.

In the early implementation phases of HRRP in FY 2013, the maximum readmission penalty was 1%, but was raised to 3% for recent years [10]. While this value seems small, the penalty is applied to all patients’ reimbursements with all conditions for the entire year, not just to readmitted patients for the six conditions. The effect is that penalties for all hospitals are $428–$528 million [15]. Many believe that reducing unnecessary hospital readmissions will reduce healthcare costs and improve the quality of care [2]. Consequently, several developed countries around the world have now formulated hospital readmission reduction policies. A systematic review of readmission policies in Denmark, England, Germany, and the U.S. highlighted each country’s policies’ advantages and disadvantages [3]. The review determined that there is not enough evidence to give recommendations on the optimal readmission policies design. However, what is clear is that hospitals and policymakers both have a shared mission to provide quality healthcare to patients. The U.S. is currently not planning any major overhauls to HRRP, so this paper focuses on how U.S. hospitals can develop care strategies that help a hospital avoid penalties while providing quality care. Avoiding penalties is often complex due to the uncertainty in the probability of readmission. Furthermore, HRRP places hospitals in competition with their peers. Effective in FY 2019, CMS began classifying hospitals into five peer groups. HRRP penalties are based on the hospital’s excess readmission ratio (ERR). An ERR is the ratio of predicted-to-expected readmissions for a given condition. HRRP then applies the penalty to hospitals whose readmission rates are worse than their peer group’s median ERR score for each condition [10].

This paper’s main objective is to develop a multi-condition model that helps hospitals identify low-cost, high-quality care strategies that are likely to avoid HRRP penalties with a hospital-defined confidence level. Despite worldwide attention on hospital readmissions, there is no consensus on optimal care strategies that will achieve readmission reduction goals. Therefore, this paper aims to determine their impact on hospitals’ actions and define care strategies using a scenario-based stochastic model. A unique contribution of this work is the model’s use of probabilistic constraints to control the stochastic readmission probabilities across all patients for each health condition. The model can be used as a tool for healthcare managers and tactical stakeholder decision-makers in response to HRRP to develop a care strategy for each health condition. Understanding the types of treatment plan decision-making helps them invest their money wisely and achieve their readmission reduction goals. Therefore, the hospital plans to use these decision-making tactics to achieve the ambitions outlined in the strategic plan to avoid the financial penalty. Finally, the model also contributes to the field as the first readmission reduction optimization model that incorporates risk into the decision-making process to avoid punitive hospital readmission reduction policies.

## 2. Literature Review

In this section, we highlight the existing hospital readmission literature that is relevant to the model in this paper. The review is organized into three sub-sections: *Statistical*, *predictive*, and *optimization* modeling approaches for reducing hospital readmissions. *Statistical* approaches analyze statistics, penalty rates, and readmission policy to improve the efficiency of this system. *Predictive* approaches aim to predict readmission rates for a specified period. *Optimization* approaches mathematically model some aspects of the readmission policy with objective functions and constraints. Our paper contributes are to the *optimization* approaches of literature for hospital readmissions. Therefore, *statistical* and *predictive* approaches are not the focus of this paper, but we will give a brief overview of the work done in these approaches.

### 2.1. Statistical Approaches

*Statistical* approaches investigate and report average hospital readmission rates and their variability. Van Walraven et al. (2011) [16] completed a systematic review of 34 studies and found the median readmission level to be 27.1%, though the percentage varied from 5% to 79%. However, Thompson et al. (2016) [17] questions the reliability of HRRP readmission estimates and claims that approximately 25% of payments for excess readmissions are tied to unreliable readmission rate estimates. Uncertainty in healthcare settings is an important and pervasive issue [18,19]. Acquiring further information through sampling can reduce this effect [20,21]. More recent statistical approaches have estimated the impact of new HRRP mechanism designs on the number of hospitals penalized and at what level they are penalized [22], and analyzed readmission rates by insurance type [23].

### 2.2. Predictive Approaches

*Predictive* approaches aim to estimate the probability of readmission. There has been extensive work in this domain, and we refer the reader to systematic reviews of prediction models [24,25,26]. Predictive models use techniques such as logistic regression, survival analysis, and machine learning. Some articles focus on specific health conditions, such as Zhong et al. (2019) [27] who developed a logistic regression model to predict readmission risks for high-risk COPD patients. Chen et al. (2019) [28] introduced readmission risk models with the incorporation of latent heterogeneity to represent superior prediction performance, recognize risk factors to target the most at-risk individuals, and evaluate multiple hospitals with composite metrics. A tree-based classification method was proposed by Shams et al. (2015) [29] to estimate the predicted probability of readmission. Finally, Horwitz et al. (2011) [30] recognized that the patient’s return behavior, or the probability of readmission, follows a normal distribution at the point of any decision epoch; we will draw on this feature in our modeling assumptions.

### 2.3. Optimization Approaches

An in-depth review of *optimization* approaches on hospital readmission can be found in Zhang et al. (2016) [6], which also shows that the HRRP policy does not always motivate hospitals to reduce readmission rates. The authors take operational and economic perspectives to analyze the effectiveness of the HRRP policy. Interestingly, they claim that competition between hospitals can increase the number of hospitals that prefer to pay penalties instead of reducing readmissions, and they develop conditions under which this preference occurs. The Latent Topic Ensemble Learning algorithm by Baechle et al. (2020) [31] designed to predict readmissions to help decision makers in hospitals. Their algorithm addressed the current system shortcomings in utilizing unstructured data from multiple hospitals and used CMS cost criteria as the metric for system performance. Alaeddini et al. (2019) [32] developed an integrated framework of risk prediction and discharge optimization to balance the early discharge risks with hospital congestion. Another integration study between optimization and prediction models is discussed in Helm et al. (2016) [33]. They worked on optimization for a post-discharge monitoring schedule and staffing plan to support monitoring needs. The new method integrated classical prediction models with machine learning and transfer learning to generate an individualized estimate of readmission time to the density function. Alvarado et al. (2016) [34] modeled the HRRP policy and formulated a penalty-incentive model for hospital readmissions in a game-theoretic setting between an insurer and a hospital. The hospital seeks to maximize its revenue while the insurer seeks to minimize its cost. They identify a win-win region for the penalty-incentive factor in which both agents are better off compared to doing nothing. Moreover, Bastani et al. (2016) [35] analyzed Medicare’s Pay-For-Performance (P4P) programs to improve patient care, using HRRP as a case study. They compared the penalty-only mechanism to symmetric penalty-incentive designs for HRRP. Later, Andritsos & Tang (2018) [36] developed the funder’s optimization model in which the patient’s readmissions can be “jointly controlled” by the efforts exerted by both the hospital and patient, and they also provide some policy guidelines on the likely effect of different reimbursement schemes like P4P and other schemes of Fee-For-Service (FFS) and Bundled Payment (BP) in the context of readmission-reduction programs. Arifoğlu et al. (2021) [37] focused on the treatment plans of a single condition and three parties of patients, hospitals, and the regulator to model the HRRP reimbursement scheme. Bastani et al. (2016) [35] is one of the first to investigate symmetric-incentive designs and Alvarado et al. (2016) [34] is one the first to investigate inequitable penalty-incentive designs for HRRP. In recent extended work, Alvarado et al. optimized an asymmetric hospital readmission reduction mechanism design in a game-theoretic setting which takes into account penalty-only policies that are optimal for the insurer in a decentralized setting but provide the worst outcome for the hospitals. However, none of these models are designed to help the hospital develop condition-specific strategies for avoiding the HRRP penalties [38].

To better explore the impact of hospitals’ actions in developing care strategies to capture the fact that readmission probabilities and targets are random parameters in the optimization models, we use *Stochastic Programming* (SP). SP as a type of optimization problem that accounts for uncertainty in which some or all of the optimization problem parameters are described by random or probabilistic variables rather than by deterministic features. Common SP techniques include two-stage and multi-stage programming, scenario construction, and probabilistic (or chance) constraints. SP models have been developed for many real-world decision-making problems, including electrical generation capacity planning [39], financial planning [40], and healthcare management [41].

The model presented in this paper uses a SP optimization approach with probabilistic constraints. Probabilistic constrained mathematical programs were introduced by [42] and have been utilized in multiple healthcare settings [43,44,45]. In our proposed scenario-based stochastic model, probabilistic constraints are introduced to provide a confidence level for meeting a target readmission rate defined by the hospital. The introduced probabilistic constraint works by selecting care strategies that keep the hospital’s mean readmission probability below the hospital’s mean peer-group readmission level (or other defined target) with a user-defined confidence level to avoid the HRRP financial penalties. Each scenario is a set of outcomes for the cost of the treatment plan and the mean probability of readmission. The decisions define a care strategy for the hospital that aims to select low-cost, high-quality treatment plans that avoid the HRRP penalty. The work proposed in this model is the first stochastic programming approach for stakeholder decision-making in response to HRRP. The model leverages assumptions about the readmission probabilities to develop a tractable and valuable care strategy model for the hospital.

## 3. Methodology

In this section, we first define the problem setting. Next, we introduce the modeling assumptions and provide a mathematical formulation. Due to the probabilistic constraints, we also present a deterministic equivalent form of the model.

### 3.1. Problem Statement

Consider a hospital that must select a care strategy for multiple health conditions over a specified decision period. Each condition has a set of *treatment plans* that can be considered for each patient. A *care strategy* is the selection of treatment plans for each patient over the hospital’s decision period. The hospital’s objectives are to select a care strategy that (a) minimizes cost, (b) provides high quality of care to patients, and (c) avoids HRRP penalties. The hospital has a target readmission rate and a confidence level in which they would like to meet its target readmission rate. Complicating the matter is that readmission probabilities are stochastic.

**Figure 1:**
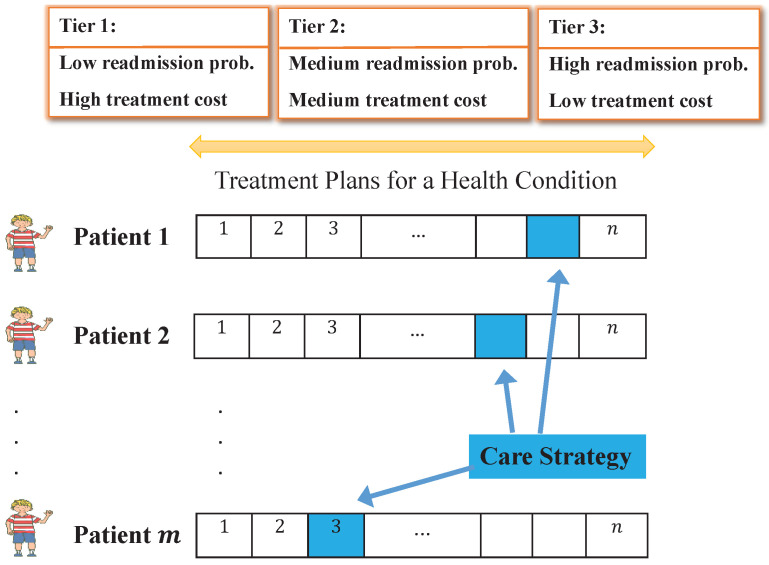
Depiction of the problem statement and the selection of a care strategy for a single condition with *m* patients and *n* treatment plans.

The illustration in Figure 1 shows an example of the care strategy for one condition. A treatment plan must be selected for each patient, and the combination of selections form the care strategy. The challenge lies in the trade-off between readmission rates and cost. For ease of reference, the treatment plans are classified into three levels in terms of readmission probability and treatment cost: Tiers 1, 2, and 3. Higher cost treatment plans are likely to be more effective and result in lower probabilities of readmission (Tier 1, left side). Lower cost treatment plans typically are less effective and have less impact on reducing the probability of readmission (Tier 3, right side). For example, medication reconciliation may be classified as Tier 3 whereas a transition coach may be classified has Tier 1. However, even the estimated readmission probability is stochastic because patients react differently to each intervention. Thus, selecting a care strategy is not a guarantee that readmission rates will be reduced to the expected rate. Tiers are not constant or standard for all hospitals; hospitals’ stakeholders decide how to distribute *n* treatment plans between these tiers for conditions. A treatment plan tiers define a patient’s history of previous treatments and the response to those treatments to reduce hospital readmission in operational treatment decisions for hospitals and specific health conditions.

### 3.2. Model Assumptions

Before presenting the model formulations, we first list several modeling assumptions and the corresponding justifications:

1. **Selecting a care strategy for a given time period**: the proposed model is a strategic decision-making policy model that would require periodic (e.g., monthly or quarterly) decisions based on changing goals, treatment plans, budgets, etc.
2. **The probability of readmission is uncertain**: historical data provides the probability of readmission based on condition, but some treatment plans have better success rates than others and some patients are more at-risk for readmission due to behavioral and health reasons.
3. **Only one treatment plan with a known cost must be chosen for each patient visit**: the modeling approach assumes that the cost of each treatment plan is fixed at the time of the decision and that exactly one treatment plan must be chosen for each patient.
4. **The percentage of hospitals penalized using the *mean readmission probability* as a target is a reasonable estimator of the percentage of hospitals penalized using the *median ERR***: recall that CMS penalizes hospitals based on ERR which is a riskadjusted ratio of the number of *predicted* readmissions at a hospital to the *expected* the number of readmissions at an average hospital over a three-year performance period. Under HRRP, if a given condition has 25 or more eligible discharges and the ERR is greater than the peer group median ERR, the hospital is subject to a penalty. Otherwise, the hospital avoids the penalty for that condition. Unfortunately, ERR scores are for hospitals over three years and are not directly associated with a treatment plan or care strategy. Therefore, we needed a direct measure to set as a target for the hospital. To solve this dilemma, we explored using readmission probabilities as an estimator. One motivation for doing so is that high ERR scores commonly correspond to high readmission probabilities. ERR measures include a risk-adjustment factor whereas the mean readmission probability does not, thus we next provide evidence of the validity of the assumption.

To test our last assumption, we introduce a *confusion matrix (CM)*, a tool commonly used to analyze the performance of binary and multi-measure methods. The HRRP data sets for each peer group are classified into two classes: Penalized and non-penalized. The actual measure assumes the current HRRP practice, which uses the median ERR to determine whether a hospital is penalized for each category. The predicted measure is determined based on whether the corresponding readmission probability is over the mean of readmission probability for the peer group. The most basic *CM* terms in this study are: *True Positive (TP)*; *False Positive (FP)* (known as a “Type I error”); *False Negative (FN)* (known as a “Type II error”); and *True Negative (TN)*.

**Figure 2:**
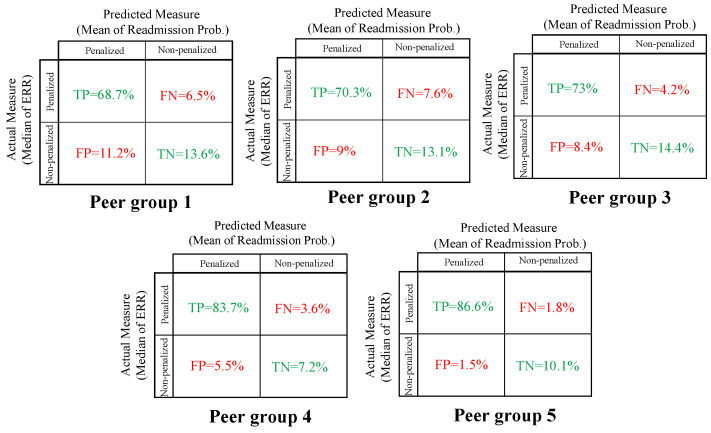
Confusion matrices of current HRRP measure (Median ERR) and predicted measure (Mean of Readmission Prob.) for all five peer groups.

The results of the *CM* are shown in Figure 2. Two of the important performance rates that are often computed from a *CM* for a binary measure are **accuracy** (e.g., how often is the measure correct?) which is computed by TP+TN, and **miss-classification rate**, or “error rate” (e.g., how often is the measure wrong?), which is computed as FP+FN. Table 1 gives an accuracy and miss-classification rate for all peer groups. In each group, the accuracy of the predicted measure is more than 82.3% for each peer group. The miss-classification rate is lowest for peer group 5 at 3.3% and highest for peer group 1 at 17.7%. Based on these performance rates in Table 1, we reasonably accept that the mean of the readmission probability is a good and fair estimate of the median of ERR. If a hospital is not confident in this assumption (e.g., a hospital in peer group 1), then recall that the hospital can adjust its confidence level or select a different target readmission rate.

### 3.3. Scenario-Based Stochastic Model

The representation and modeling of uncertainty as a crucial stage in SP can be different based on the specific SP techniques used. Scenario representation of uncertainty is one of the most commonly used techniques in SP. The main idea of the scenario representation is to generate a large number of scenarios where each scenario represents a possible realization of underlying uncertain factors. This method is an approximation of the true distribution of the uncertainties. Scenarios are generated to simulate the uncertainty of the readmission probability and the treatment plan costs. Uncertainty is represented in terms of random experiments with outcomes denoted by ω. The set of all outcomes is represented by Ω. Let *C* be a set of conditions for which a single hospital wants to select a care strategy and $P^{c}$ be the set of patients with a condition c∈C that the hospital expects to treat in the decision period. A set of $K^{c}$ treatment plans for each patient exists. A probability p(ω) is assigned to each scenario such that $\sum_{\omega \in \Omega }p\left(\omega \right)=1$. Each treatment plan *k* for patient *p* in condition *c* under scenario ω has a cost of $a_{pk}^{c}\left(\omega \right)$ and mean readmission probability of $\mu_{pk}^{c}\left(\omega \right)$. For each condition *c*, the hospital will set a target readmission rate of $\theta^{c}$ with a confidence level of $\beta^{c}$. The binary decision variable $x_{pk}^{c}\left(\omega \right)$ in our stochastic model shows the chosen treatment plans for patients in each condition under scenario ω. All the sets, parameters, and variables used in the model are summarized in Table 2.

In the uncertain and real environments, hospitals need to determine a target readmission rate $\theta^{c}$. In HRRP, it is challenging for a hospital to predict whether they will be penalized because this depends on its ranking in the peer group. However, there exists a care strategy $x^{c}\left(\omega \right)=\sum_{p\in P^{c}}\sum_{k\in K^{c}}x_{pk}^{c}\left(\omega \right)$ with uncertain mean readmission probability $\mu^{c}\left(\omega \right)=\sum_{p\in P^{c}}\sum_{k\in K^{c}}\mu_{pk}^{c}\left(\omega \right)$ that satisfies the probabilistic constraint of $P\left(f\left(\mu^{c}\left(\omega \right)x^{c}\left(\omega \right)\right)\leq \theta^{c}\right)\geq \beta^{c}$. The $\beta^{c}$ is a confidence level determined by decision makers at the hospital. For instance, given $\beta^{c}$, the decision makers then need to determine a feasible target readmission rate $\theta^{c}$ such that care strategy $x^{c}\left(\omega \right)$ satisfies $P\left(f\left(\mu^{c}\left(\omega \right)x^{c}\left(\omega \right)\right)\leq \theta^{c}\right)\geq \beta^{c}$, where the expected readmission probability across all patients for condition *c* will be less than $\theta^{c}$ with a chance of at least $\beta^{c}$. Note that $\beta^{c}$ and $\theta^{c}$ will need to be selected such that the constraint yields a feasible care strategy $x^{c}\left(\omega \right)$.

The scenario-based care strategy Stochastic Model (SM) is presented in problem (1)–(4). The objective function (1) minimizes the expected treatment plan cost and the expected penalty for the hospital by choosing confidence level vector $\vec{\beta }$. Constraint (2) requires exactly one treatment plan to be selected for each patient. Constraint (3) is a probabilistic constraint for each condition, which requires that the expected mean readmission probability of the selected treatment plans across all patients of condition c∈C be less than or equal to the target readmission rate $\theta^{c}$ with a confidence level of $\beta^{c}$. Constraint (4) specifies the decision variable as a binary constraint.

Note that the probabilistic Constraint (3) in the SM formulation complicates the formulation and thus, we develop the deterministic equivalent form of the problem in the next sub-section.

(1) $$\begin{array}{cc} \mathrm{SM}\;=\;\mathrm{Min} & \sum_{\omega \in \Omega }\left(p\left(\omega \right)\sum_{c\in C}\sum_{p\in P^{c}}\sum_{k\in K^{c}}\left(a_{pk}^{c}\left(\omega \right)x_{pk}^{c}\left(\omega \right)\right)\right)+\mathrm{EP}\left(\vec{\beta }\right) \end{array}$$

(2) $$\begin{array}{cc} s.t.\;\; & \sum_{k\in K^{c}}x_{pk}^{c}\left(\omega \right)=1,\;\forall p\in P^{c},c\in C,\omega \in \Omega \end{array}$$

(3) $$\begin{array}{cc} \; & P\left(\frac{\sum_{p\in P^{c}}\sum_{k\in K^{c}}\mu_{pk}^{c}\left(\omega \right)x_{pk}^{c}\left(\omega \right)}{|P^{c}|}\leq \theta^{c}\right)\geq \beta^{c},\;\forall c\in C,\omega \in \Omega \end{array}$$

(4) $$\begin{array}{cc} \; & x_{pk}^{c}\left(\omega \right)\in \left\{0,1\right\},\;\forall p\in P^{c},k\in K^{c},c\in C,\omega \in \Omega . \end{array}$$

The expected penalty $\mathrm{EP}(\vec{\beta })$ represents the penalty cost if the care strategy fails to avoid the HRRP penalty. For simplification, we estimate this as a constant term based on average treatment costs. For each condition *c*, the probability of the care strategy failing is $\left(1-\beta^{c}\right)$. Thus, we estimate the expected penalty $\mathrm{EP}(\vec{\beta })$ for confidence level vector $\vec{\beta }$ as:

(5) $$\begin{array}{cc} \; & \mathrm{EP}\left(\vec{\beta }\right)=\sum_{c\in C}\left(1-\beta^{c}\right)\left(R^{c}Penalty\right), \end{array}$$

where $R^{c}$ is the proportion of the penalty cost contributed by condition *c* is defined by:

(6) $$\begin{array}{cc} \; & R^{c}=\frac{|P^{c}|{\bar{a}}^{c}}{\sum_{\hat{c}\in C}\left|P^{\hat{c}}\right|{\bar{a}}^{\hat{c}}}. \end{array}$$

Penalty is the hospital’s annual penalty cost (e.g., the hospital’s penalty cost in dollars for FY 2018) and ${\bar{a}}^{c}$ is the mean treatment cost for condition *c*. Note that the probabilistic constraint (3) in the SM formulation complicates the formulation and thus we develop the deterministic equivalent form of the problem in the next sub-section.

### 3.4. Deterministic Equivalent Model

Probabilistic constraints provide a relatively robust approach, but can be complicated to solve. The literature shows that patient return behavior (e.g., probability of readmission) follows the Gaussian (or Normal) distribution [30] when it is not time-dependent. Fortunately, this assumption of Gaussian readmission rates enables the development of a deterministic equivalent model. The probabilistic constraint (3) has binary variables $x_{pk}^{c}\left(\omega \right)$ and random parameters $\mu_{pk}^{c}\left(\omega \right)$, which follow a continuous distribution. The expected readmission probabilities are independent and non-identically distributed Gaussian random parameters, $\mu_{pk}^{c}\left(\omega \right)∼N\left({\hat{\mu }}_{pk}^{c}\left(\omega \right),\lambda_{c}{\hat{\mu }}_{pk}^{c}\left(\omega \right)\right)$ for $\lambda_{c}>0$, which provides for a deterministic equivalent model [46,47]. Here $\lambda_{c}$ is the coefficient of variation for condition *c*. This combination yields the following theorem.

**Theorem** **1.** *For condition c∈C and under each scenario ω∈Ω, consider $|P^{c}\left|*\right|K^{c}|$ random parameters $\mu_{pk}^{c}\left(\omega \right)∼N\left({\hat{\mu }}_{pk}^{c}\left(\omega \right),\lambda_{c}{\hat{\mu }}_{pk}^{c}\left(\omega \right)\right)$, $\lambda_{c}>0$, and ${\hat{\mu }}_{pk}^{c}\left(\omega \right)$. Then, if $x_{pk}^{c}\left(\omega \right)\in \left\{0,1\right\}$ for each $p\in P^{c}$ and $k\in K^{c}$, the following constraints are equivalent:*

$$\begin{array}{c} P\left(\frac{\sum_{p\in P^{c}}\sum_{k\in K^{c}}\mu_{pk}^{c}\left(\omega \right)x_{pk}^{c}\left(\omega \right)}{|P^{c}|}\leq \theta^{c}\right)\geq \beta^{c}\Leftrightarrow \sum_{p\in P^{c}}\sum_{k\in K^{c}}{\hat{\mu }}_{pk}^{c}\left(\omega \right)x_{pk}^{c}\left(\omega \right)\leq \mu_{c}^{*}\left(\omega \right), \end{array}$$

where $\mu_{c}^{*}\left(\omega \right)$ is the unique root of:

(7) $$\begin{array}{c} \theta^{c}\left|P^{c}\right|-\mu_{c\omega }\left(x\right)=\sqrt{\lambda_{c}\mu_{c\omega }\left(x\right)}*\phi^{-1}\left(\beta^{c}\right), \end{array}$$

*where $\mu_{c\omega }\left(x\right)=\sum_{p\in P^{c}}\sum_{k\in K^{c}}{\hat{\mu }}_{pk}^{c}\left(\omega \right)x_{pk}^{c}\left(\omega \right)$ and ϕ is the cumulative distribution of the standard normal distribution.*

**Proof.**  Recall that if $\mu_{pk}^{c}\left(\omega \right)$ are independent Gaussian with mean ${\hat{\mu }}_{pk}^{c}\left(\omega \right)$ and variance $\lambda_{c}{\hat{\mu }}_{pk}^{c}\left(\omega \right)$:

The left-hand side of probabilistic constraint (3) is equal to:

(8) $$\begin{array}{ccc} LHS & = & P\left(\frac{\sum_{p\in P^{c}}\sum_{k\in K^{c}}\mu_{pk}^{c}\left(\omega \right)x_{pk}^{c}\left(\omega \right)}{|P^{c}|}\leq \theta^{c}\right)\\ & = & P\left(\sum_{p\in P^{c}}\sum_{k\in K^{c}}\mu_{pk}^{c}\left(\omega \right)x_{pk}^{c}\left(\omega \right)\leq \theta^{c}\left|P^{c}\right|\right)\\ & = & P\left(N\left(0,1\right)\leq \frac{\theta^{c}\left|P^{c}\right|-\mu_{c\omega }\left(x\right)}{\sqrt{\lambda_{c}\mu_{c\omega }\left(x\right)}}\right) \end{array}$$

where $\mu_{c\omega }\left(x\right)=\sum_{p\in P^{c}}\sum_{k\in K^{c}}{\hat{\mu }}_{pk}^{c}\left(\omega \right)x_{pk}^{c}\left(\omega \right)$.

Then the original probabilistic constraint (3) becomes:

(9) $$\begin{array}{ccc} P\left(\frac{\sum_{p\in P^{c}}\sum_{k\in K^{c}}\mu_{pk}^{c}\left(\omega \right)x_{pk}^{c}\left(\omega \right)}{|P^{c}|}\leq \theta^{c}\right)\geq \beta^{c} & \Leftrightarrow & \frac{\theta^{c}\left|P^{c}\right|-\mu_{c\omega }\left(x\right)}{\sqrt{\lambda_{c}\mu_{c\omega }\left(x\right)}}\geq \phi^{-1}\left(\beta^{c}\right)\\ & \Leftrightarrow & \mu_{c\omega }\left(x\right)\leq \mu_{c}^{*}\left(\omega \right)\\ & \Leftrightarrow & \sum_{p\in P^{c}}\sum_{k\in K^{c}}{\hat{\mu }}_{pk}^{c}\left(\omega \right)x_{pk}^{c}\left(\omega \right)\leq \mu_{c}^{*}\left(\omega \right) \end{array}$$

because the left-hand side of inequality (9) is decreasing in $\mu_{c\omega }\left(x\right)$, where $\mu_{c}^{*}\left(\omega \right)$ is the unique root of the equation:

(10) $$\begin{array}{cc} \; & \theta^{c}\left|P^{c}\right|-\mu_{c\omega }\left(x\right)=\sqrt{\lambda_{c}\mu_{c\omega }\left(x\right)}*\phi^{-1}\left(\beta^{c}\right). \end{array}$$

□

A summary of the additional notation for the deterministic equivalent model is provided in Table 3. By replacing the probabilistic constraint with its equivalent constraint, the SM from problem (1)–(4) becomes the Deterministic Equivalent Model (DEM) given in problem (11)–(14):

(11) $$\begin{array}{cc} \mathrm{DEM}\;=\;\mathrm{Min}\; & \sum_{\omega \in \Omega }\left(p\left(\omega \right)\cdot \sum_{c\in C}\sum_{p\in P^{c}}\sum_{k\in K^{c}}\left(a_{pk}^{c}\left(\omega \right)x_{pk}^{c}\left(\omega \right)\right)\right)+\mathrm{EP}\left(\vec{\beta }\right) \end{array}$$

(12) $$\begin{array}{cc} s.t.\;\; & \sum_{k\in K^{c}}c_{pk}\left(\omega \right)=1,\;\forall p\in P^{c},c\in C,\omega \in \Omega \end{array}$$

(13) $$\begin{array}{cc} \; & \sum_{p\in P^{c}}\sum_{k\in K^{c}}\mu_{pk}^{c}\left(\omega \right)x_{pk}^{c}\left(\omega \right)\leq \mu_{c}^{*}\left(\omega \right),\;\forall c\in C,\omega \in \Omega \end{array}$$

(14) $$\begin{array}{cc} \; & x_{pk}^{c}\left(\omega \right)\in \left\{0,1\right\},\;\forall p\in P^{c},k\in K^{c},c\in C,\omega \in \Omega . \end{array}$$

## 4. Model Demonstration

This section provides a numerical demonstration of DEM for two real hospitals. We first describe the data sets for each hospital and then discusses the design of experiments and computational results.

### 4.1. Design of Experiments

CMS publishes HRRP supplemental data every fiscal year for more than 3200 hospitals and health centers across the U.S. The data specifies the peer group classifications, the number of cases, and ERR for each hospital for all six conditions over a 3-year period [10]. For our study, real hospital data was collected from CMS for the FY 2018 for all six HRRP conditions to demonstrate numerical examples of the developed model. CMS uses a 3-year performance history; hence, the FY 2018 dataset contains results from July 2013 to June 2016 [10]. The peer groups are defined based on the percentage of the dual proportion which is the number of patients dually eligible for Medicare and full-benefit Medicaid during the fiscal year. Hospitals in the first peer group have the lowest dual proportion and hospitals in the 5th peer group have the highest dual proportion. We randomly selected hospitals in peer group 2 for our analysis. To demonstrate the model’s value under different extremes, we separated the hospitals into two categories: *High-* and *low-performing* hospitals, respectively, with 0–2 and 3–6 penalized conditions. Note that the number of hospitals with no penalized conditions refers to hospitals that are already doing better than the mean peer group in all six health conditions. A total of 58% of hospitals in the peer group 2 belong to the low-performing hospital group and 42% of them belong to the high-performing hospital group.

Two hospitals were selected from peer group 2 based on the number of penalized conditions, one from the low-performing hospital group (Hospital “A”) and one from the high-performing hospital group (Hospital “B”). Due to privacy issues, we will refrain from mentioning the name of the hospitals. Hospital “A” in New York, USA is penalized for five of six HRRP conditions and currently receives a cumulative 1.35% penalty. Hospital “B” in California, USA is penalized for one of six HRRP conditions and currently receives a cumulative 0.2% penalty. The probability of readmission and penalties for each hospital are given in Table 4, where “P” appears to indicate which conditions were penalized for the respective hospitals. For each condition, the second peer group’s mean readmission probability was used as the target readmission rate; the presumed values are also listed in Table 4. We aim to identify a three-month care strategy for each hospital based on its risk level. The number of patients in three months for each hospital for each condition is given in Table 5. The number of treatment plans can be determined by the opinion of physicians or management. Based on the opinion of our physician collaborator, 10 treatment plans per patient for each condition have been selected in this study. These 10 plans have been distributed as follows: Tier 1 has three treatment plans (*k* = 1,2,3), Tier 2 has four treatment plans (*k* = 4,5,6,7), and Tier 3 has three treatment plans (*k* = 8,9,10). The known cost information of conditions is collected from [48]. This website uses the hospital’s zip code to provide three cost figures: Below the fair price, fair price, and highest price. The fair price is defined as the price that you should reasonably expect a medical service to cost if you shop for care [48]. The triangular distribution (TRIA) was used to generate the cost information and these parameters are also summarized in Table 5. The value of β varies in the design of experiments between 50–98%.

To estimate the number of scenarios, *n*, necessary for estimating a population mean with (1−α)100% confidence and error no larger than ϵ is:

(15) $$\begin{array}{cc} \; & n>\frac{\left(z_{\frac{\alpha }{2}}^{2}\right){\hat{s}}^{2}}{\epsilon^{2}}, \end{array}$$

where ${\hat{s}}^{2}$ is a decent estimate of the population variance based on a reduced number of scenarios and *z* is the standard normal distribution [49]. The total care cost (objective function) over 25 scenarios yielded a variance of ${\hat{s}}^{2}=\$156,721.15$ for Hospital “A” and ${\hat{s}}^{2}=\$81,246.64$ for Hospital “B”. By applying formula (15) with 99% confidence and ϵ=150, we chose to round up to |S|=50 scenarios for this study.

We use the normal distribution to generate the readmission probability $N({\hat{\mu }}_{pk}^{c}\left(\omega \right),\lambda_{c}{\hat{\mu }}_{pk}^{c}\left(\omega \right))$ assuming the coefficient of variance $\lambda_{c}$ = 0.25 for all conditions. More specifically, for the 50 scenarios, the mean readmission probability of each hospital for each condition was taken from CMS data [10] (as shown in Table 4) and used to generate the mean readmission rate for each patient based on the normal distribution. Then, for each patient and condition, we additionally generated $|K^{c}|$ readmission probabilities for each treatment plan. Generating the costs of treatment plans followed the same process based on the triangular distributions from Table 5 for each condition.

### 4.2. Computational Results

In this section, we report the experimental results of experiments for two selected hospitals to show DEM performance under 50 scenarios. The hospitals’ current situation has been considered to validate the results of the developed model. Baseline testing validates and compares the performance of DEM with the current hospital situation as a known standard of reference. The optimal care strategies for selected hospitals are explained in three experiments.

1. **Cost of optimal care strategies for varying levels of β**

**Figure 3:**
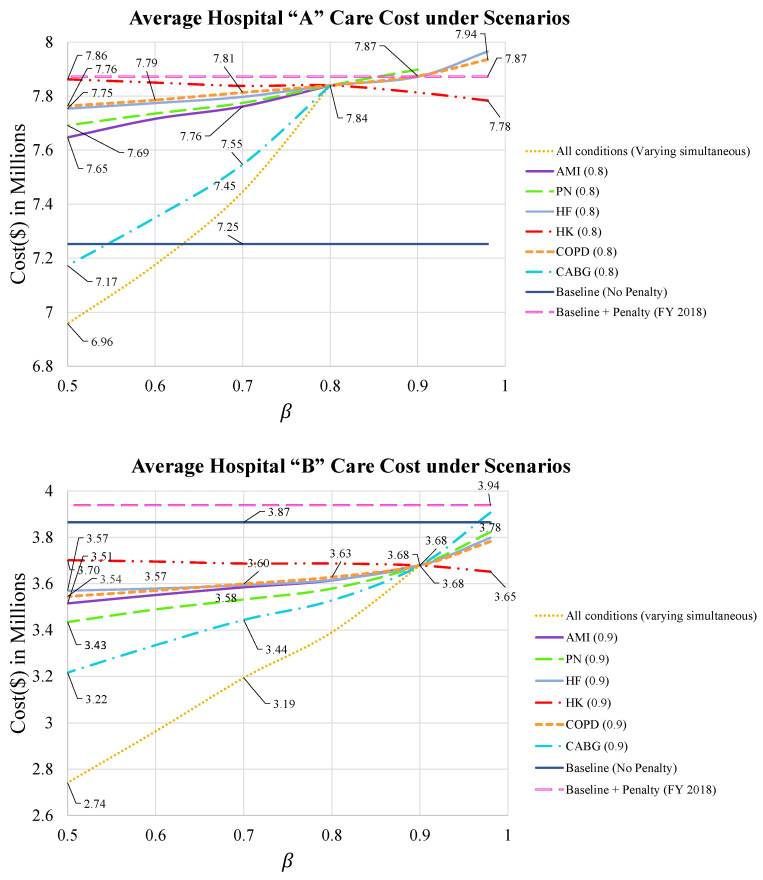
Average expected cost of care strategy for hospital “A” and “B” under scenarios.

The cost of optimal care strategies considering the expected penalty based on varying levels of β for both hospitals is shown in Figure 3. Each point reported in Figure 3 corresponds to the average optimal cost of care **plus** the expected penalty by choosing confidence level of $\beta^{c}$ for each condition c∈C. The “Baseline (No Penalty)” line (solid, dark blue line) shows the hospital’s cost if HRRP did not exist and there were no penalties, which is computed as the sum-product of the mean cost and the number of patients across the six conditions as shown in Equation (16). Additionally, the “Baseline + Penalty (FY 2018)” line (dashed, pink line) shows the hospital’s current costs with HRRP penalties over the same time period as computed by Equation (17). These two lines display the hospital’s current situation for total cost care and consider validating the decisions from the model. Thus, any decisions below the dashed, pink line would yield cost savings and improvement for the hospital.

(16) $$\begin{array}{cc} \; & \mathrm{Baseline}\;(\mathrm{No}\;\mathrm{Penalty})=\sum_{c\in C}\left|P^{c}\right|{\bar{a}}^{c} \end{array}$$

(17) $$\begin{array}{cc} \; & \mathrm{Baseline}\;+\;\mathrm{Penalty}=\sum_{c\in C}\left|P^{c}\right|{\bar{a}}^{c}+Penalty. \end{array}$$

Other than “Baseline (No Penalty)” and “Baseline + Penalty (FY 2018)” lines in Figure 3, each line shows the average expected the total cost of care for the hospital when β varies along the *y* axis. The line labeled “All conditions” is the total cost for dropping $\beta^{c}$ simultaneously for all six conditions. The highest feasible $\beta^{c}$ for the “All conditions” line will be referred to as $\beta^{*}$ where the average expected cost of care converges. The condition-specific lines (e.g., AMI(0.8) for hospital “A”) indicate that the $\beta^{c}$ value was varied for that condition (e.g., AMI) while the other five conditions remained fixed at $\beta^{*}$ (e.g., 80%).

For hospital “A”, the AMI, PN, and CABG conditions have the highest feasible $\beta^{c}$ at 0.8, 0.9, and 0.8, respectively, which can be seen where the condition-specific lines stop at these points. Thus, $\beta^{*}=80\%$ becomes the highest feasible β in the “All Conditions” line. The “All conditions” line generally has the lowest cost for most values of $\beta^{c}$ because it takes less money to attain these lower standards for all conditions simultaneously. hospital “A” can feasibly reduce its cost from the “Baseline+Penalty” level by selecting a care strategy with β equal to 0.8 for all conditions. However, they would need to select $\beta^{c}>0.64$ for all conditions to achieve the pre-HRRP cost levels (e.g., where “All conditions” intersects the “Baseline (No Penalty)” level). This analysis is valuable to “hospital A” because they can determine how much additional money it would require to improve its confidence level and shift its ranking in the peer group to avoid HRRP penalties for that condition. This can be done simultaneously across all conditions by considering different strategies for each condition.

For the higher-performing hospital “B”, the AMI condition has the highest feasible $\beta^{c}$ at 0.9. Thus, $\beta^{*}=90\%$ is the highest feasible β in hospital “B” for the “All Conditions” line. For hospital “B”, most solutions are below both the “Baseline (No Penalty)” and the “Baseline + Penalty” lines because the hospital was already doing well pre-HRRP for five of six conditions, so the penalty cost was very small. Even selecting a care strategy with β at the maximum feasible value of 90% has a lower cost than the pre-HRRP level by nearly $260,000.

Since the mean readmission probability of both hospitals for HK is already much lower than the mean readmission probability in the second peer group, the cost of care slowly decreases and shows less expected penalty for this condition. Baseline lines are considered to display the need for improvement in the following hospitals and the developed model can help them with that. In Figure 3, hospital “A” has high penalties with the “Baseline + Penalty” line at ($7.87 million) and needs improvement. Most solutions for both hospitals “A” and “B” result in penalties below the hospitals’ current situation “Baseline + Penalty”, indicating how valuable the developed model can be in helping them identify areas of improvement. Even for hospital “B”, the results from the model mark improvement. On a final note, recall from Table 5 that CABG is the most expensive condition therefore, targeting this condition for reduced care levels is cost-effective for both hospitals. For example, dropping the confidence level for CABG from 0.8 to 0.7 or 0.6, but maintaining high confidence level standards at $\beta^{*}=80\%$ for all other conditions can bring significant cost-savings to hospital “A”.

2. **Mean hospital readmission probability for the optimal care strategies, given by condition**

The left-hand side of constraint (13) measures the mean hospital readmission probability for each condition and scenario. Given the optimal solution $x_{pk}^{{*}^{c}}\left(\omega \right)$ then Equation (18) measures average optimal mean readmission rates through all scenarios:

(18) $$\begin{array}{cc} \; & {\bar{\mu }}_{c}^{*}\left(\omega \right)=\sum_{\omega \in \Omega }p\left(\omega \right)\cdot \left(\sum_{p\in P^{c}}\sum_{k\in K^{c}}\mu_{pk}^{c}\left(\omega \right)x_{pk}^{{*}^{c}}\left(\omega \right)\right),\;\forall c\in C. \end{array}$$

**Figure 4:**
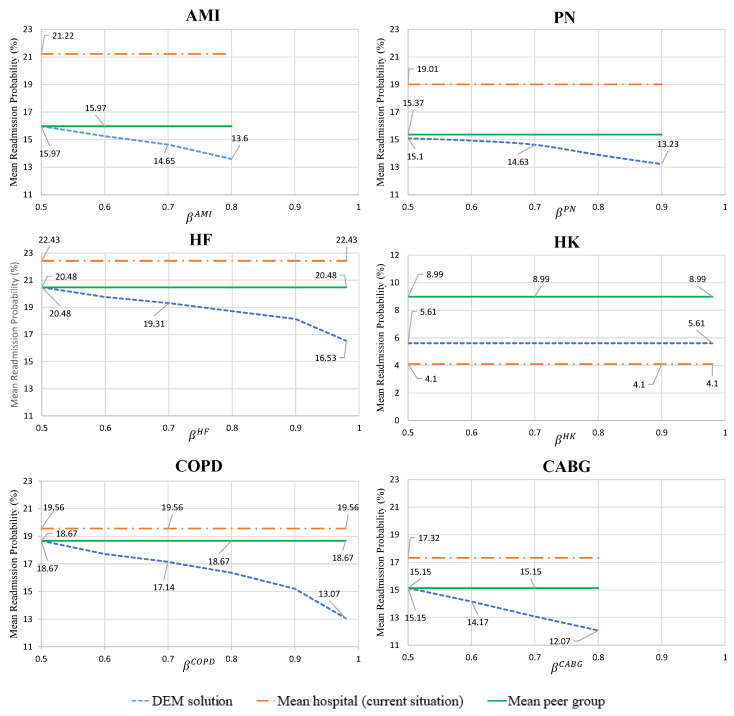
Hospital “A” mean readmission probability for the optimal care strategy under scenarios (DEM solution) compared to its current rate, and the peer group mean for each condition.

**Figure 5:**
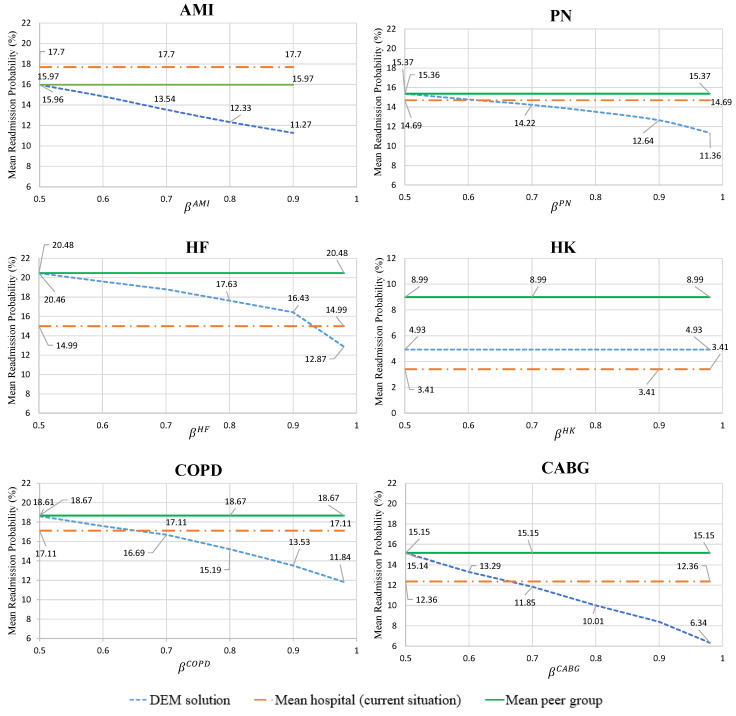
Hospital “B” mean readmission probability for the optimal care strategy under scenarios (DEM solution) compared to the its current rate and its peer group mean for each condition.

Figure 4 and Figure 5 depict the readmission probabilities for each hospital and condition for varying levels of β through all scenarios. If the mean readmission probability for any health condition is greater than the mean peer group, the hospital will have financial penalties for the upcoming year. To avoid the financial penalty, the hospital’s goal is to keep their mean readmission probabilities below the mean readmission level of their peer group. The introduced probabilistic constraints in the model provides a confidence level for achieving this goal. The line labeled “DEM Solution” is the average optimal mean readmission rates across 50 scenarios (${\bar{\mu }}_{c}^{*}\left(\omega \right)$) for the specific condition shown. The “Mean peer group” line refers to the mean readmission probability of the second peer group for the specific condition, which was the assumed target. Finally, the “Mean hospital (current situation)” line is the mean readmission probability of the hospital without the impact of HRRP. When the “Mean hospital (current situation)” line is above the “Mean peer group” target, the condition is penalized. To validate the computational results from this experiment, any decision below the “Mean hospital (current situation)” line would yield improvement for the hospital and reduce financial penalties. For decisions above the target line based on the selected confidence level, there is still a place for improvement from the hospital. Therefore, these results show how the developed model can propose care strategies for hospitals to help them avoid the financial penalty by selecting treatment plans for each condition. Whenever the mean readmission probabilities from the “DEM solution” is below the “Mean hospital (current situation)”, then the hospital has the opportunity to decrease their readmission probabilities to avoid the HRRP financial penalties.

Since hospital “A” received a penalty for five of six conditions, the “Mean hospital” lines are above the “Mean peer group” lines for all but the HK condition. As a result, HK maintains a constant (or flat) line for the “DEM solution” because the mean readmission probability of hospital is already much lower than the mean readmission probability in the second peer group. For the other five conditions (AMI, PN, HF, COPD, and CABG), the “DEM solution” has an inverse relationship between β and the mean readmission probability. This observation makes sense because high β values indicate high quality-of-care levels and low readmission rates. COPD, HF, and CABG have the steepest slopes for the “DEM solution”, indicating that they are cost-effective investments for the care strategy of hospital “A”.

Since hospital “B” received a penalty for only one of six health conditions, the “Mean hospital” line is above the “Mean peer group” line for this one AMI condition. For the HK condition in this hospital, the “Mean hospital” readmission probability is significantly lower than the “Mean peer group” readmission rate. Therefore, its “DEM solution” line is flat and is worse than the “Mean hospital” line because relaxing standards for HK is still much better than the mean of the peer group. However, the other four conditions (PN, HF, COPD, and CABG) have an intersection point between the “DEM solution” and the “Mean hospital” lines. After this intersection point (e.g., higher β values), any solution indicates that the DEM solution is an improvement over the current mean readmission probability for the hospital. Therefore, the hospital can have fewer penalties and even avoid the financial penalty for the next upcoming year when their mean readmission probability passes the intersection point. AMI, COPD, and CABG have the steepest “DEM solution”, indicating that they are the most cost-effective investments for the care strategy of hospital “B”.

3. **Analysis of the decisions for the optimal care strategies, given by condition**

**Figure 6:**
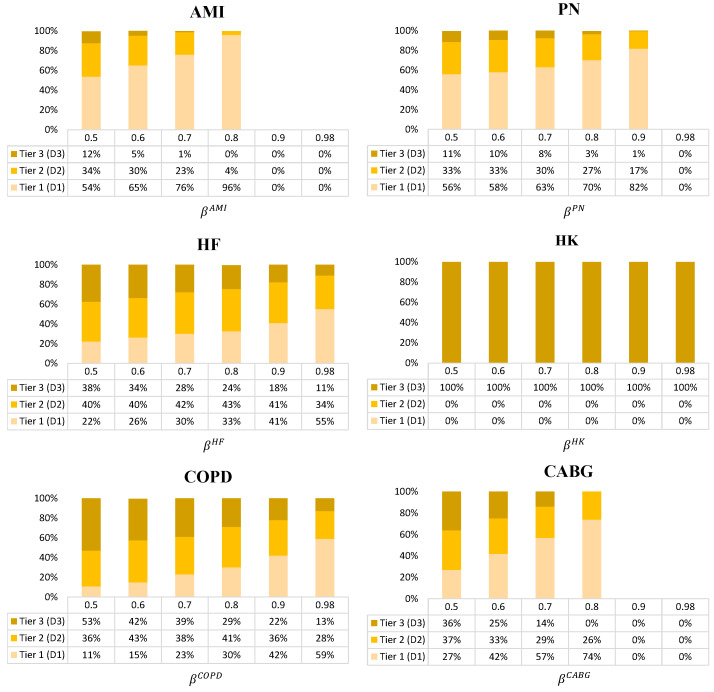
Care strategy for hospital “A” for each condition across all scenarios (Tier 1: Low probability and high cost, Tier 2: Medium probability and cost, and Tier 3: High probability and low cost).

**Figure 7:**
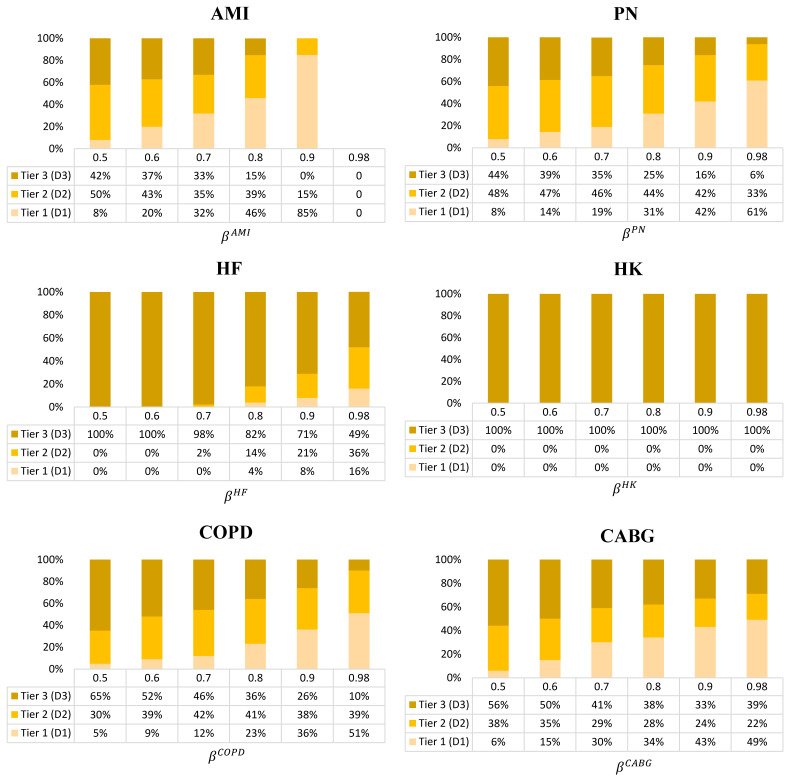
Care strategy for hospital “B” for each condition across all scenarios (Tier 1: Low probability and high cost, Tier 2: Medium probability and cost, and Tier 3: High probability and low cost.

The average optimal care strategies based on varying levels of β for hospitals “A” and “B” across all scenarios are shown in Figure 6 and Figure 7, respectively. As an example care strategy for hospital “A”’s AMI condition, the 50% confidence level indicates the selection of Tier 1 treatment plans for 54% of patients, Tier 2 treatment plans for 34% of patients, and Tier 3 treatment plans for 12% of patients. Following this care strategy, hospital “A” has a 50% confidence level that they will hit the target value and avoid the HRRP penalty. If they want to increase the confidence level to 70%, then they should shift to a care strategy that elects 76% of Tier 1 treatment plans, 23% of Tier 2 treatment plans, and 1% of Tier 3 treatment plans. Correspondingly, the costs will go up (see Figure 3 for AMI), but the readmission probability will drop (see Figure 4 for AMI) and they will be more likely to achieve the target readmission rate.

In general, as β increases, the percentage of Tier 1 treatment plans increases while the percentage of Tier 3 treatment plans decreases. Since the expected readmission probability of the HK condition for both hospitals is already much lower than the target readmission rate, the recommended care strategy is always to use Tier 3 treatment plans.

It is recommended that hospital “A” place a high investment in Tier 1 treatment plans (low-probability and high-cost) to achieve its target value. It is especially true for AMI, PN, HF, and CABG which correspond to the largest gap between the hospital’s mean readmission probability and the peer group mean. In general, hospital “B” can more easily keep a balance between all three tiers for PN, COPD, and CABG. However, hospital “B” should select the most Tier 1 treatment plans for the AMI condition.

## 5. Discussion

As managerial insights, the Stochastic Model (SM) and data analysis presented in this paper provide several contributions to healthcare management science. First, SM is a practical tool for hospitals to develop care strategies that avoid the HRRP penalty. The SM model minimizes treatment costs under multiple scenarios without sacrificing care quality through strategic selection of care strategies and treatment plans to reduce readmission rates. By reducing readmission rates below the peer group rates, the hospital avoids financial penalties. The developed scenario-based model provides a valid approximation for uncertainty in the problem. Zhang et al. (2016) [6] claimed that competition between hospitals can be counterproductive and increases the number of hospitals, which prefer paying penalties over reducing readmissions. However, SM provides an opportunity for hospitals to reduce their readmission rates in a constructive competition. Therefore, the hospital can use SM to develop a care strategy that keeps its readmission rate competitive with its peer group under a user-specified target and confidence level.

Second, the computational results, based on real data from CMS, shows that SM provides this opportunity for the hospital to select low-cost, high-quality treatment plans that avoid the HRRP penalties for its peer group. The analysis results indicate that healthcare managers and stakeholder decision-making can use SM to optimize their hospital’s care strategy. The introduced probabilistic constraints in the model provides a confidence level for meeting a target readmission rate that can also set decision-makers as the goal for healthcare centers. Patient care experience is also improved since the probabilities of returning to the hospital for the next 30 days after discharge are decreased. Moreover, selecting minimum cost treatments lowers the total care cost for hospitals and out-of-pocket expenses for patients, which results in more patient satisfaction. Regarding hospital insights, most health systems and hospitals are doing well managing readmission probabilities for HK, indicating policymakers should select Tier 3 treatments. However, CABG is the most expensive condition among health conditions; targeting CABG or other costly conditions for reduced care levels is cost-effective and policymakers should focus more on CABG Tier 1 treatments.

Third, it is important to note that the developed model is for tactical decision-making, not operational decision-making. Therefore, operational decisions such as considering treatment time, receptivity to medication, or equipment availability are not focused on these levels of decisions. However, the treatments can be a combination of treatments such as medications (adding or changing the doses), follow up after discharge, etc. Furthermore, this policy would require periodic (e.g., monthly or quarterly) review based on changing goals, treatment plans, costs, budgets, etc., to make the best long-term, or strategic, decision.

Last but not least, readmission rates at hospitals are a critical measure of their service levels and substantially impact their costs. The developed SM can be used for holistic or condition-specific strategies to reduce HRRP penalties for the hospital. Therefore, hospital managers can use the model to recognize the conditions where more improvement is needed, which is an advancement upon other recent literature focusing on treatment plans of a single health condition [37]. Some studies have also investigated only one of the six health conditions for hospital readmissions [27,50]. The SM model provides an opportunity to efficiently allocate resources to health conditions with higher readmission probabilities than the mean peer group to reduce the risk of readmission. Moreover, the model brings cost reduction due to the improved resource allocation and spending cost in conditions with higher readmission levels. We compare the current policy to the new model on five categories: Decision-making approach, treatment plan selection, treatment plans cost, stochastic data, and patient experience. Table 6 summarizes operations of current readmission reduction policy and the modeling approach developed in this paper. The proposed SM has a few limitations, such as the assumption of normal readmission probabilities in Section 3.4 to develop DEM. The data analysis also assumed the treatment plan costs were triangular for demonstration purposes. However, hospitals already have their own cost data and can implement this feature within the model without these primary restrictions for input data to the model.

## 6. Summary and Future Research

A scenario-based stochastic programming model with probabilistic constraints was developed and solved to obtain the hospital’s optimal care strategy for avoiding HRRP penalties. The hospital selects a target readmission rate and a confidence level in which they would like to meet its target readmission rate. The model solutions enable the hospital to minimize the expected treatment care cost without sacrificing the quality of care, measured by the reduced probability of readmission. By using the proposed model, hospitals can identify care strategies for each of the six HRRP conditions to prevent the financial HRRP penalty in the coming years. The trade-off between the cost of care, reduced readmission rates, and confidence levels for the care strategies across all conditions were explored in this study. Model outputs will be valuable to hospitals as they examine the expected cost of hitting its target, the expected improvement to its readmission rates, and a synopsis of the care strategy across different tiers of treatment plans.

To demonstrate the value and output of the scenario-based stochastic programming model, we use real data for two hospitals implementing the tactical planning model over a three-month period. Results indicate that highly penalized, or low-performing, hospitals should invest more in Tier 1 (low-probability and high-cost) treatment plans to achieve their target. Meanwhile, the hospitals with fewer penalties, or high-performing, can more easily invest in Tier 2 and Tier 3 plans, especially for conditions where their current readmission rates are much better than the target readmission rate. Additional analysis of the trade-off between readmission probabilities and expected costs of treatment plans provided insight into which conditions could prove to be the most cost-effective investments.

In future research, we would like to extend the model to a multi-stage model to account for the three-year gap performance history for penalty assessments. Furthermore, we would like to incorporate the models into agent-based simulations or hybrid simulation models to account for competition among hospitals in the same peer group to investigate optimal HRRP policy design.

## Figures and Tables

**Table 1:** Performance rates of current HRRP median ERR measure and mean of readmission probability.

Peer Group	Accuracy	Misclassification Rate
1	82.3%	17.7%
2	83.4%	16.6%
3	87.4%	12.6%
4	90.9%	9.1%
5	96.7%	3.3%

**Table 2:** Notation for the stochastic programming model.

Sets	
*C*:	Set of conditions, indexed by *c*
Ω:	Set of scenarios, indexed by ω
$K^{c}$:	Set of treatment plans with condition c∈C, indexed by *k*
$P^{c}$:	Set of patients with condition c∈C, indexed by *p*
**Parameters**	
p(ω):	Probability of scenario ω∈Ω
$\theta^{c}$:	Target readmission rate for condition c∈C, given as a probability between 0 and 1
$\beta^{c}$:	Confidence level to satisfy the probability constraint for condition c∈C
$\vec{\beta }$:	Confidence level vector $\vec{\beta }=\left(\beta^{1},\beta^{2},\ldots ,\beta^{c}\right)$
$a_{pk}^{c}\left(\omega \right)$:	Cost of treatment plan k∈K for patient $p\in P^{c}$ with condition c∈C under scenario ω∈Ω
$\mu_{pk}^{c}\left(\omega \right)$:	Mean readmission probability of treatment plan k∈K for patient $p\in P^{c}$ with condition c∈C under scenario ω∈Ω
$\mathrm{EP}(\vec{\beta })$:	Expected penalty for confidence level vector $\vec{\beta }$
$R^{c}$:	Proportion of baseline cost contributed by condition c∈C
${\bar{a}}^{c}$:	Mean cost of treatment plans for condition c∈C
*Penalty*:	One-year penalty cost for the hospital
**Decision variable**	
$x_{pk}^{c}\left(\omega \right)$:	$x_{pk}^{c}\left(\omega \right)=1$ if treatment plan k∈K for patient $p\in P^{c}$ with condition c∈C under scenario ω∈Ω is selected, $x_{pk}^{c}\left(\omega \right)=0$ otherwise

**Table 3:** Additional notation for the deterministic equivalent model.

Additional Notations	
$\lambda_{c}$:	Coefficient of variance for the Gaussian random variable for condition c∈C
$\mu_{c\omega }\left(x\right)$:	$\sum_{p\in P^{c}}\sum_{k\in K^{c}}{\hat{\mu }}_{pk}^{c}\left(\omega \right)x_{pk}^{c}\left(\omega \right)$
$\mu_{c}^{*}\left(\omega \right)$:	Unique root of Equation (10)
ϕ:	Cumulative distribution of the standard normal distribution N(0,1)

**Table 4:** Mean readmission probability in the second peer group, mean readmission probability, and penalized conditions for selected hospitals.

		Hospital “A”		Hospital “B”	
**Cond.**	**Peer Group 2 Mean** **Readmission Prob.**	**Mean Readmission** **Prob.**	**P**	**Mean Readmission** **Prob.**	**P**
AMI	15.97%	21.21%	×	17.69%	×
PN	15.37%	19.01%	×	14.69%	
HF	20.48%	22.43%	×	14.99%	
HK	8.01%	4.21%		3.41%	
COPD	18.67%	19.56%	×	17.11%	
CABG	15.15%	17.33%	×	12.36%	

P: Penalized conditions.

**Table 5:** Average number of patients in 3 months and the cost distributions for the selected hospitals.

	Hospital “A”		Hospital “B”	
**Cond.**	**No.** **Patients**	**Cost($) Distributions** **(TRIA)**	**No.** **Patients**	**Cost($) Distributions** **(TRIA)**
AMI	52	(6745, 8431, 21,078)	18	(8439, 10,548, 26,370)
PN	132	(6312, 7890, 19,725)	69	(7897, 9871, 24,678)
HF	126	(5990,7487, 18,718)	44	(7494, 9367, 23,418)
HK	96	(3585, 9359, 31,814)	76	(4196, 9001, 25,542)
COPD	47	(6460, 8075, 20,188)	22	(8082, 10,102, 25,255)
CABG	21	(39,864, 56,297, 161,385)	7	(47,382, 67,664, 196,103)

**Table 6:** Comparison chart between operations of current hospital readmission policy and the scenario-based stochastic programming model.

Current Readmission Reduction Policy	VS	Scenario-Based Stochastic Programming Model
Decentralized focus on health conditions	**Decision-making** **approach**	Centralized focus on each health condition
Scattered decisions for patients	**Treatment** **plan** **selection**	Optimal care strategy based on holistic approach for multiple patients and conditions
Not formally considered	**Treatment** **plans cost**	Selecting treatment with minimum cost
Not formally considered	**Stochastic data**	Included via probabilistic constraints
High readmission probability, then more hospital visits and higher out-of-pocket expenses	**Patient** **experience**	Lower readmission probability, then less hospital visits and lower out-of-pocket expenses

## Data Availability

The datasets analyzed during the current study are publicly available in the CMS repository [10], https://www.cms.gov accessed on 24 March 2021, and Healthcare Bluebook repository [48], https://www.healthcarebluebook.com accessed on 15 April 2021.
